# Reduction of Dislocations of the Femur without the Aid of Pulleys

**Published:** 1853-02

**Authors:** 


*Reduction of Dislocations of the Femur without the aid of Pulleys.*—In an article by Dr. John Watson, in a late number of the New York Medical Times, we find the following remarks on the reduction of dislocation of the femur without the aid of pulleys. While we are glad to see that Dr. Reid's plan has been successfully applied in the New York Hospital, we regret to notice that the tenor of the remarks by Dr. W. does injustice to Dr. Reid. The writer refers to several German surgeons in such a way as to leave the impression that the subject had very little novelty in this country at the time Dr. Reid's article appeared, and that Dr. R. is not entitled to any credit on the score of originality. There seems to be an indefinite traditionary notion that Dr. Nathan Smith recommended a plan somewhat similar to that described so perspicuously, and demonstrated so satisfactorily by Dr. Reid. The description of Dr. Smith's method, if we are correctly informed, by his son, in the memoirs prepared by the latter, is not very clear, and the method as described is not very practicable. At all events, it never was adopted to any extent in this country. Dr. Reid, however, was cognizant of the tradition, and is careful to say so in his essay. As respects the German authors who have "carefully studied and systematised" the subject, we wish Dr. Watson had been more explicit. It strikes us as rather remarkable that the fruits of the thorough investigation of the matter in Germany were not better known among us prior to the present time, if they have been in progress of development since 1823! Will not some one conversant with German medical literature, give the American public an analysis of what has been accomplished in Germany relative to the subject during the last thirty years, of which we have been so strangely ignorant, subjecting patients, in the mean time, to the barbarous treatment by pulleys, which are now ascertained to be useless!

The following is the quotation referred to:

Surgery, as well as medicine, has its fashions; and while we are ready to apply the extension pulleys to new uses, we are learning to dispense with them in some of the very accidents for the cure of which they were originally introduced. Our celebrated countryman, Nathan Smith, about the commencement of the present century, as I learn from one of his early pupils, Dr. Batchelder, of this city, as well as from the memoirs of him prepared by his son, Dr. N. R. Smith—was in the habit of pointing out to his students a method of reducing luxations of the femur on the dorsum of the ilium by merely flexing, adducting or abducting, and rotating, the thigh upon the pelvis. Others, both in this country and Europe, have, since the time of Nathan Smith, been accidentally successful in a few similar cases. In Germany, since 1823, the subject has been more carefully studied and systematized by Colombat, Wattmann, Kluge and Rust; and in our own state, within the past year, by Dr. W. W. Reid, of Rochester, whose process, as described in the last volume of the Transactions of the New York State Medical Society, is nearly identical with that of the latter writer. I am happy to say that the attention which Dr. Reid has anew attracted to this subject, is likely to make the flexing, adducting, abducting and rotating process, the general mode of treatment in all recent luxations of the head of the femur backward; and that, at the New York Hospital, two, if not three cases have already been reduced, without the aid of pulleys, by this method.

Hoping that these suggestions may be further tested, I remain, with becoming respect, Yours, &c.,

New York, December 18, 1852. JOHN WATSON.

We find in the Jan. No. of the American Journal of Med. Sciences, the following notice of reduction of a luxated femur, at Boston, Mass.:

"Dr. Parkman reduced a recent dislocation of the hip very easily with his hands alone, and unassisted, the patient being thoroughly etherized. The head of the bone was in the ischiatic notch. Taking the foot in his hand, Dr. P. bent the leg on the thigh, and the thigh on the abdomen, and, with slight outward rotation of the foot, drew the limb downward, with immediate reduction of the displaced bone. The dislocation was caused by the fall of some bags of coffee upon the patient who was aiding in raising them from a vessel's hold."

